# Epidemiology of severe heart disease among Unified Health System (SUS) users in Rio Grande do Norte: a cross-sectional study

**DOI:** 10.1590/1516-3180.2023.0261.R1.07032025

**Published:** 2025-07-04

**Authors:** Gustavo Gomes Torres, Angelo Giuseppe Roncalli

**Affiliations:** I Programa de Pós-Graduação em Saúde Pública, Universidade Federal do Rio Grande do Norte (UFRN), Natal, Rio Grande do Norte, Brazil.; II Departamento de Odontologia, Programa de Pós-Graduação em Saúde Pública, Universidade Federal do Rio Grande do Norte (UFRN), Natal, Rio Grande do Norte, Brazil

**Keywords:** Epidemiology, Heart disease, Cardiac pacing, artificial, Unified Health System, Access to health services, Brazilian National Health Service, Pacemakers, Clinical profile

## Abstract

**BACKGROUND:**

Severe heart disease has high prevalence, morbidity, and mortality rates.Heart stimulation is important in the final stages of heart disease. The concentration of procedures in a service allows for the epidemiological analysis of our population.

**OBJECTIVE:**

To analyze the epidemiological profile of severe heart disease in Rio Grande do Norte by registering all patients undergoing artificial cardiac stimulation (ACS) in a Unified Health System reference service in Rio Grande do Norte.

**DESIGN AND SETTING:**

This cross-sectional study included all patients who underwent ACS procedures at the Hospital Onofre Lopes, Universidade Federal do Rio Grande do Norte (UFRN), from 2006 to 2021. Sociodemographic characteristics, procedures, and health conditions were examined. Additionally, a spatial analysis of casuistry was performed according to the municipality of origin.

**METHODS:**

This cross-sectional study analyzed data derived from patients treated at Hospital Onofre Lopes, UFRN, from 2006 to 2021, including sociodemographic characteristics, procedures, and health conditions.

**RESULTS:**

A total of 894 patients (male, 59.8%; mean age: 65.5 years) were included. Third-degree atrioven tricular block was indicated in 191 patients, an ischemic etiology was found in 269 patients, whereas dyspnea was reported by 398 patients. Furthermore, 69.5%, 24.4%, and 31.7% of patients had hypertension, diabetes, and dyslipidemia, respectively. Spatial analysis showed no significant differences in the formation of clusters.

**CONCLUSIONS:**

The characteristics of the service contributed to possible differences in the literature. The spatial distribution of severe heart disease was random in the state, indicating an adequate distribution of reference services even in the absence of a defined flowchart for such services.

## INTRODUCTION

 Severe heart disease has a high prevalence and incidence, with a substantial impact on morbid ity and mortality in general population. Low investment in health, inadequate access to care, and insufficient follow-up services at the primary or tertiary level are potential risk factors. ^1^ In addition to medications, patients require support in the final stages of heart disease, and artifi cial cardiac stimulation (ACS) is necessary for treating heart failure (HF).^2,3^

 In the Unified Health System (SUS), procedures are performed in public or private referral centers. Complex procedures performed for severe heart disease are restricted to some centers, usually public, according to a specific ordinance by the Ministry of Health.^4^ In Rio Grande do Norte, complex procedures are performed in this period exclusively at the Hospital Universitário Onofre Lopes (HUOL), Universidade Federal do Rio Grande do Norte (UFRN). Thus, the HUOL focuses on all SUS patients undergoing procedures, specifically those with severe heart disease. Therefore, this study represented practically all SUS patients in Rio Grande do Norte who under went complex procedures related to severe heart disease. Data on this population in our country are scarce. In Brazil, few centers portray their populations based on epidemiological patterns. 

 Universal access to health services is not only a constitutional guarantee and a pillar of the SUS but also an expression of the right to citizenship. Citizenship has a supervisory and action role with the potential for concrete results, change, and improvement in care. Access to health services is a transformative tool of reality. ^5^

 Spatial analysis can describe the characteristics and patterns existing in geographical spaces for a given factor and establish relationships among different variables quantitatively.^6^ It is a fundamental instrument in public health that enables the devel opment of technologies for data analysis in a geographical space. A detailed study on the health situation and its trends allows for the identification of variables that reveal the social, economic, and environmental structure of the environment. ^7^

 Access to specialized public health service by the entire population, irrespective of their social condition or place of origin, is a premise and obligation of the SUS. This has not been previously evaluated in relation to ACS in Rio Grande do Norte. Given the group of highly complex procedures and the absence of well-defined flowcharts for access to the reference service, spatial analysis can aggregate informa tion for health managers and the scientific community. Additionally, the analysis of this sample establishes an epidemiological profile that characterizes an SUS referral service and provides an estimate of the prevalence of severe heart disease in a state in Northeastern Brazil. 

## OBJECTIVE

 The current study primarily aimed to evaluate the epidemiological profile of SUS patients in a referral service for ACS in Rio Grande do Norte from 2006 to 2021. The specific objectives were (i) to analyze the characteristics of the population served in relation to demographic and epidemiological peculiarities, use of medications, and particularities of procedures and (ii) to descriptively evaluate the spatial distribution of patients served based on the municipality of origin. 

## METHODS

 This quantitative, analytical, cross-sectional retrospective study was conducted at the HUOL, a reference center for ACS by the SUS in Rio Grande do Norte. Data were collected from primary sources, and medical records were analyzed. All patients under going ACS procedures in this service from 2006 to 2021 were included, totaling 894 patients. 

 Data were obtained by reviewing the medical records based on the institution’s list of procedures. The analysis was performed using physical records requested from a specific sector. In cases of nonlocation, electronic medical records were searched. Because the sample comprised all procedures performed during that period, there were no exclusion criteria. 

 For the sociodemographic profiles, data related to age and sex were evaluated. The variables assessed in the epidemiological profile were divided into four groups—namely, (i) epidemiological characteristics (residence, underlying disease, symptomatology); (ii) comorbidities (systemic arterial hypertension, diabetes, kidney failure, dyslipidemia, HF, smoking, catheterization changes, valvular heart disease); (iii) use of medications (amiodarone, angiotensin-converting enzyme [ACE] inhibitors, digoxin, diuretic, acetylsalicylic acid [ASA], spironolactone, beta-blockers); and (iv) characteristics of the procedures (electrocardiographic alteration, procedure, need for temporary pacemaker, implanted device, stimulation mode, and complications). 

 Data were stored in Microsoft Excel spreadsheets and were analyzed via Stata, a statistical software for data science, to establish the epidemiological profile. Categorical variables were expressed as absolute values and relative frequencies, whereas continuous variables were presented as measures of central tendencies. 

 For the spatial analysis, the rate was established based on the number of procedures per 100,000 inhabitants in the municipality of origin. Thematic maps were prepared using QGIS software, and univariate spatial analysis was performed with Global Moran’s I index using GeoDa software to verify the level of spatial interde pendence between the analysis units (municipalities). 

 A reporting guide for observational studies (STROBE Statement) was used. Data collection was initiated after the approval of the project by the Research Ethics Committee of UFRN (opinion number: 4.880.641) on August 3, 2021. 

## RESULTS

 From 2006 to 2021, 894 ACS procedures were performed at the HUOL-UFRN. Among the patients treated, 533 were male (59.8%). The patients’ mean age was 63.8 years for males (median, 65 years) and 68.1 years for females (median, 69 years), with age extremes ranging from 13 to 100 years. 

 Regarding the patients’ region of origin, more than half came from the metropolitan region of Natal, Rio Grande do Norte. The predominant underlying etiology was ischemia, which was detected in 269 (30.6%) patients. Chagas disease, valvular heart disease, and other etiologies were identified in 98 (11.2%), 98 (11.2%), and 28 (3.2%) patients, respectively. A causal etiology was not established in 421 (47.9%) patients. 

**Figure 1:**
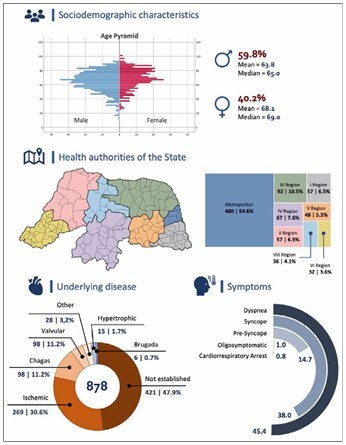
Infographic shows the demographic and epidemiological characteristics of the sample. Rio Grande do Norte, 2021.

 Presyncope or syncope was the main complaint in 462 patients (52.7%), among whom 38% and 14.7% had syncope and presyncope, respectively. Dyspnea was reported in 398 (45.4%) patients. Only 7 (0.8%) patients presented with a cardiorespiratory arrest event, and symptomatology was not reported in 9 (1%) patients (Figure 1). 

**Figure 2:**
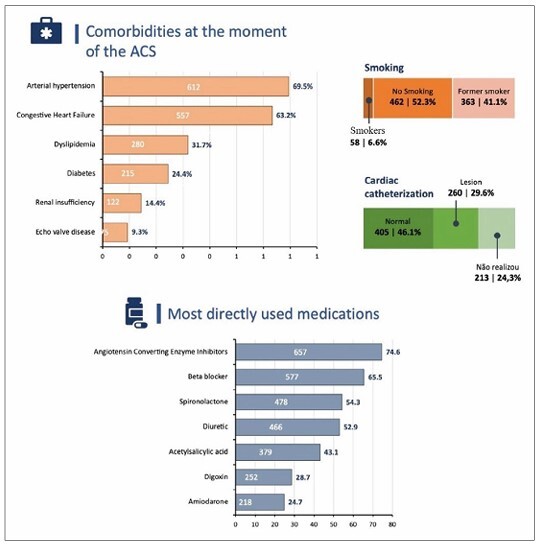
Infographic illustrates the clinical characteristics, medications, and risk factors. Rio Grande do Norte, 2021.

 With respect to related comorbidities, systemic arterial hyper tension occurred in 612 patients (69.5%), congestive HF in 557 (63.2%), dyslipidemia in 280 (31.7%), diabetes mellitus in 215 (24.4%), renal failure in 122 (14.4%), and valvular heart disease on Doppler echocardiography in 75 (9.3%). Regarding smoking, 462 (52.3%) patients denied current or previous smoking, 58 (6.6%) were smokers, and 363 (41.4%) reported having stopped smoking for at least 5 years. Obstructive lesions in the coronary arteries were verified at catheterization in 260 patients (29.6%), including 213 who did not undergo the examination (Figure 2). 

 The most directly used medications with cardiovascular effects were ACE inhibitors or angiotensin receptor blockers in 657 patients (74.6%), beta-blockers in 577 (65.5%), spironolactone in 478 (54.3%), diuretics in 466 (52.9%), ASA in 379 (43.1%), digoxin in 252 (28.7%), and amiodarone in 218 (24.7%). 

 Electrocardiographic alterations indicative of the procedure were left bundle branch block in 245 patients with HF (27.4% of the sample), followed by third-degree atrioventricular (AV) block in 191 patients (21.4%). A total of 163 (18.3%) patients underwent generator replacement. Tachycardia events determined the need for the procedure in 121 (13.5%) patients. 

 The most commonly performed procedure was device implantation (*n* = 610, 68.6%), followed by generator replacement due to battery exhaustion and/or electrode dysfunction (*n* = 218, 24.5%) and other types of approaches (*n*= 61, 6.9%). 

 According to the type of device used, 346 (39.1%) patients required a conventional pacemaker implant, 292 (33%) patients required atriobiventricular pacemaker (cardiac resynchronization therapy [CRT]) implants, and 154 (17.4%) patients required a double-chamber implantable cardioverter-defibrillator (ICD). ICD with CRT, which was the most complex prosthesis, was used in 94 (10.6%) patients. 

 Dual-chamber stimulation, stimulating the atrium and ventricle(s), corresponded to the mode in 810 (90.8%) patients, with only ventricular stimulation in 76 (8.5%) patients and only atrial stimulation in three (0.3%) patients. In the three procedures, the system was completely removed without device replacement (0.3%). 

**Figure 3:**
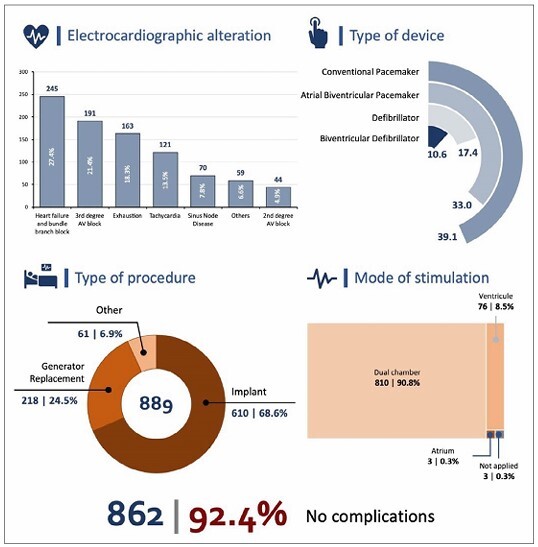
Infographic shows the technical characteristics related to ACS procedures. Rio Grande do Norte, 2021.

 The complication rate was 7.6% (Figure 3). The mean left ventricular ejection fraction (LVEF) was 33.6% (median, 30%). The mean creatinine level was 1.04 mg/dL (median, 1 mg/dL). The mean serum potassium level was 4.19 mg/dL (median, 4.6 mg/dL). 

 Regarding the patients’ origin, the majority (*n* = 384) of patients originated from Natal, whereas 47 and 38 patients were from Parnamirim and Mossoró, respectively. Of the 168 municipalities, 110 referred patients during the study period. 

**Figure 4:**
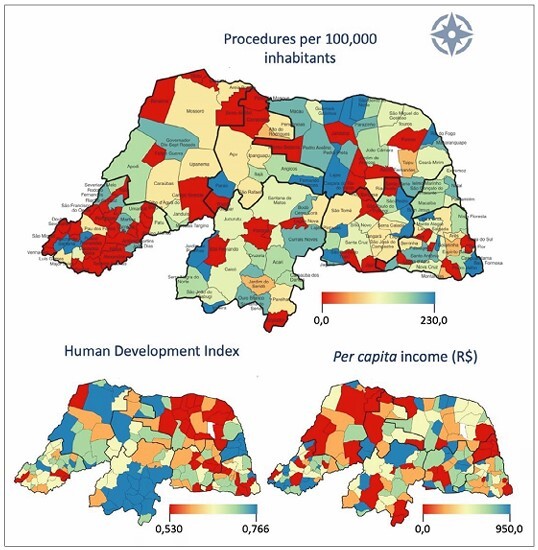
Infographic of thematic maps for the distribution of procedures, Human Development Index, and per capita income. Rio Grande do Norte, 2021.

 Based on the population of municipalities in 2014, the rate of pro cedures per 100,000 inhabitants was evaluated (Figure 4) to estimate the prevalence of severe heart disease.^7^ Natal (the capital city) appeared in position 22, with a rate of 44.55, whereas Mossoró (the second largest city in the state) was only in position 91, with a rate of 13.37. ^7^

 As for spatial influence, the Global Moran’s I index value obtained was 0.046, without statistical significance, indicating no spatial pattern in the distribution of procedures per 100,000 inhabitants. The distribution of socioeconomic variables (Human Development Index and *per capita* income) was also quite different from the rate of procedures per inhabitant (Figure 4), suggesting the absence of socioeconomic determination. 

## DISCUSSION

 Severe heart disease is associated with complex medical pro cedures. Despite potentially fatal situations, medical technol ogy can support and improve the patients’ quality of life. The most complex ACS procedures encompass this universe almost entirely. As the HUOL is the only service in the state dedicated to complex procedures, we consider this record of patients under going ACS to be a portrait of severe heart disease within the SUS in Rio Grande do Norte. 

 The Brazilian Register of Cardiac Pacemakers, Defibrillators, and Resynchronizers (BRCP) is a national database established to collect data on cardiac pacemakers, defibrillators, and resynchronizers; such an obligation stems from ordinance number 41 of March 1994 by the Ministry of Health. ^8^ While its completion depends on the ini tiative of the team, the obtained data allow for the establishment of a procedural profile in Brazil. On August 1, 2008, the Ministry of Health, by ordinance number 1,559, instituted the National Policy for Regulation of the SUS, which determines the competences of fed erative entities. Among other obligations of the municipality are to guarantee access to the referenced population according to agreed and integrated programming and to act in an integrated manner at the State High Complexity Regulation Center (CERAC).^9^

 Comparing our data with the results of analysis conducted by Pachón et al., ^10^ based on the BRCP, we observed a higher percentage of ischemic etiology, dyspnea as a complaint, lower degree of sinus node disease, and second-degree AV block. These differences can be explained by the higher profiles of patients undergoing CRT in our sample. The lower profile of ischemic patients in the aforementioned study might have been underestimated by the use of the Registro Brasileiro de Marcapassos (RBM) as a database. RBM data were analyzed from 2000 to 2014, with a focus on the num ber of procedures and their distribution by region in the country. ^11^

 Analysis with an active search of data from 2018 and 2019 in a cardiological reference center did not include patients under going complex procedures. ^12^ In this study, females were slightly predominant (51.8%), with a mean age of 72.9 years. These data contrast with our sample as well as with the etiological findings of Chagas disease and ischemia. The sensitivity and existence of less invasive serological investigations may explain these differences. 

 In the international context, Spain has published annual reports on ACS procedures since 1997. The analysis was performed with a focus on technical aspects, ^13^ the most recent of which coincides with our data regarding male predominance, syncope as a causal factor, and the percentage of first implants and generator changes. Differences were observed in the occurrence of ischemic etiology, HF indicative of the procedure, and the percentage of biventricular stimulation or resynchronizers. 

 The Italian Association of Arrhythmology published data for 2018. ^14^ With more than 400 centers, the population of this registry presents a higher age group, with the data being close to our findings. In 2011, we had data records of the holders of ACS devices in France,^15^ with data similar to ours in relation to the type of device used. Other centers such as those in Denmark present periodic epidemiological records with a greater focus on implanted devices and procedural complications. ^16^ Recently, the Danish registry dem onstrated similar results with regard to epidemiological data. ^17^

 The Biopace registry selected patients from 2003 to 2007 in Europe and Australia. ^18^ Compared to our data, this population had a higher mean age and proportion of males. We observed the same proportion of patients with ischemic disease, as well as a high proportion of AV block and tachycardia. The characteristics of our service, including the selection of patients for CRT and the absence of emergency care, may explain these differences. 

 National data with a clinical focus are reported by analyzing spe cific comorbidities, such as hypertrophic heart disease ^19^ and Chagas disease. ^20,21^ Other centers report clinical analyses but with a lower volume of procedures.^22-24^ A Nigerian study showed single-center data, with a smaller volume of patients. ^22^ Casola Crespo et al. ^24^ pres ent data from a Cuban center, with a higher number of procedures, but without data on CRT and ICD. They reported a higher preva lence of ventricular pacemakers than double-chamber pacemakers 

 Dubernet et al. ^25^ show data from Chile, focusing on the proce dure with a predominance of conventional pacemakers. Aktoz et al.^26^ evaluated the influence of gender and demographic data on the type of device used between 2006 and 2016. Equality was observed in relation to sex, with a mean age higher than that in our study. The proportion of ICD use in relation to pacemaker use is consistent with our findings. The high prevalence of ventricular devices (single chamber) in this study differs from general findings and our sample. 

 Khanal et al. ^27^ conducted a study on the influence of gender on device type. The percentage of single-chamber systems was higher than that reported in the general literature and in our population. The data from our study in relation to the percentage of ventricular pacemaker implants agreed with the national sample, with the majority of patients receiving double-chamber stimulation. 

 Symptoms associated with resynchronizer implants were prevalent in our study. These points are explained by the service being referenced without a gateway to emergencies, which require the most non-complex procedures that are carried out mostly in the private network. 

 The representativeness of older individuals was similar to that reported in the literature, with advanced age being a predictive factor for the need for ACS. The slight predominance in male individuals is corroborated by the literature, which shows that sex is not a relevant factor in the emergence of these pathologies. We observed a high incidence of ischemic etiology, probably because the study was limited to a tertiary school hospital with access to invasive diagnostic technologies. The lower proportion of emergency procedures also contributed to a greater etiological investigation. Dyspnoea was highlighted by a higher proportion of complex procedures related to resynchronization. 

 Clinical conditions such as hypertension and diabetes were predominant in our study population, reinforcing the role of deterioration of the conduction system and cardiac function associated with these risk factors. High use of medications such as ACE inhibitors, spironolactone, and beta-blockers is associated with the prevalence of HF. 

 Among the basic electrocardiographic changes, our profile explains the Left Branch Block as the main change, because it is required for resynchronization. 

 The HUOL at UFRN is a tertiary referral hospital for SUS patients in Rio Grande do Norte. It serves the entire state with a varied profile of patients with different complexities and severities. The characteristics of the service may explain the differences between our sample and those of other centers and services. The hospital does not provide emergency care, with all demands for ACS procedures being referenced from other centers. The availability of pacemaker procedures in affiliated hospitals associated with a lack of emergency care explains the lower proportion of less complex procedures. As procedures of greater complexity are offered only in the HUOL, their proportion in relation to conventional pacemakers increases. We then obtained a picture of almost the entire population of the state subjected to resynchronizer and defibrillator implantations using the SUS. 

 Spatial evaluation was considered to have no impact when analyzing the distribution of patients by municipality of origin. We were unable to establish clear clusters of greater access to services by merely analyzing geographical aspects (proximity to the service), regions with greater purchasing power, or regions where access to medical services is easier. Geographical differences, social inequalities, and socioeconomic differences are not reflected in greater accessibility. This result indicates homogeneous accessibility despite the absence of an established flowchart. 

 In the 1988 Constitution, health was guaranteed as a universal right of the state for all citizens. Despite these advances, we live with an unequal and exclusionary reality. Legal guarantees were an important stage in the construction of the SUS; however, in practice, thisis still a daily struggle for health professionals. To realize the right to health, we need a social model based on “human solidarity and social equality.” ^28^ Unfortunately, a “selective, focused and exclusion ary” access is observed. The challenge of upholding the constitution continues. The implementation of equitable access requires differ ent actions, depending on the social segment and clinical situation. 

 T he social situation excludes a part of the population, which is not always perceived by the public. Often, when these demands are perceived, managers lack data for an in-depth analysis; to formulate public policies to mitigate the problem. ^29^ The challenging context of the economic crisis that has set in the last decade is not only local, but also shows a series of counterpoints, such as the existence of barriers to users, such as queues for appointment and service. ^30, 31^

 The absence of a flowchart may be a factor responsible for this absence of long waiting for the procedure. Although the eligible population was restricted to patients with more severe heart disease, the lack of identification by health professionals in their gateway services may be a limiting factor for our demand. This did not prevent patients from accessing the most varied locations. We observed that the patients had access to other care flows, often through direct medical contact with our services. 

 The apparent equity detected in access did not seem comfortable. We consider it necessary and urgent that these data be made available to managers to demonstrate the importance of this population, the service itself, and the need to develop defined flows to better identify these patients, allow access, and avoid the loss of patients who could benefit from these important tools. 

 Ultimately, this group of patients corresponded to the profile of patients in our state with more severe heart disease, which is representative of the prevalence of severe heart disease in Rio Grande do Norte. Analysis of the data provides useful information for developing better health policies for patients with serious heart disease. 

## CONCLUSION

 This population corresponded to the profile of patients with advanced heart disease, which is representative of the prevalence of severe heart disease in Rio Grande do Norte. Evaluation of the clinical and epidemiological profiles of this sample showed a pattern compatible with the literature, with a higher proportion of patients with HF and a high number of complex procedures. We believe that the characteristics of the service contributed to the differences in our population in relation to the findings in the literature. 

 Despite the absence of a flowchart, the spatial distribution was not statistically significant with respect to the municipality of origin. The results should be shared with the public health managers of the state to collaborate in the elaboration of better health policies, focusing on providing patients with universal access to this important tool for heart disease treatment. 
